# Logic programming-based Minimal Cut Sets reveal consortium-level therapeutic targets for chronic wound infections

**DOI:** 10.1038/s41540-024-00360-6

**Published:** 2024-04-02

**Authors:** Maxime Mahout, Ross P. Carlson, Laurent Simon, Sabine Peres

**Affiliations:** 1 Université Paris-Saclay, CNRS, Laboratoire Interdisciplinaire des Sciences du Numérique, 91405 Orsay, France; 2 Department of Chemical and Biological Engineering, Center for Biofilm Engineering, Microbiology and Immunology, Montana State University, Bozeman, MT 59717 USA; 3 Bordeaux-INP, Université Bordeaux, LaBRI, 33405 Talence Cedex France; 4 UMR CNRS 5558, Laboratoire de Biométrie et de Biologie Évolutive, Université Claude Bernard Lyon 1, 69100 Villeurbanne, France; 5 INRIA Lyon Centre, 69100 Villeurbanne, France

**Keywords:** Software, Antimicrobials, Biochemical networks, Computer modelling, Target identification

## Abstract

Minimal Cut Sets (MCSs) identify sets of reactions which, when removed from a metabolic network, disable certain cellular functions. The traditional search for MCSs within genome-scale metabolic models (GSMMs) targets cellular growth, identifies reaction sets resulting in a lethal phenotype if disrupted, and retrieves a list of corresponding gene, mRNA, or enzyme targets. Using the dual link between MCSs and Elementary Flux Modes (EFMs), our logic programming-based tool *aspefm* was able to compute MCSs of any size from GSMMs in acceptable run times. The tool demonstrated better performance when computing large-sized MCSs than the mixed-integer linear programming methods. We applied the new MCSs methodology to a medically-relevant consortium model of two cross-feeding bacteria, *Staphylococcus aureus* and *Pseudomonas aeruginosa*. *aspefm* constraints were used to bias the computation of MCSs toward exchanged metabolites that could complement lethal phenotypes in individual species. We found that interspecies metabolite exchanges could play an essential role in rescuing single-species growth, for instance inosine could complement lethal reaction knock-outs in the purine synthesis, glycolysis, and pentose phosphate pathways of both bacteria. Finally, MCSs were used to derive a list of promising enzyme targets for consortium-level therapeutic applications that cannot be circumvented via interspecies metabolite exchange.

## Introduction

*Staphylococcus aureus* and *Pseudomonas aeruginosa* are opportunistic pathogens commonly associated with the skin microbiome and water sources, respectively. The two problematic bacteria are responsible for an estimated 1+ million deaths yearly due in part to widespread antimicrobial resistance^1^. *S. aureus* and *P. aeruginosa* are frequently co-isolated from chronic wounds and cystic fibrosis lungs^2,3^. Their interactions such as metabolite cross-feeding^4,5^ have been associated with higher resiliency to antibiotics and worse patient outcomes^6,7^. The complex nature of their interactions with other bacteria and the environment has motivated a growing number of studies involving consortia of these pathogenic bacteria, whether it is through in vivo and in vitro models^8,9^, or in silico models^10,11^. Better informed and therefore more effective intervention strategies for treating *S. aureus* and *P. aeruginosa* infections could save millions of lives and billions of dollars in healthcare expenses.

Constraint-based metabolic modelling (CBM) is a well-established systems biology field involving the computational reconstruction and analysis of biological mechanisms at multiple levels^12^. At its core are metabolic networks, hypergraphs described by a set of metabolites and reactions linked to each other by stoichiometric coefficients stored in a stoichiometric matrix. The constraint-based modelling approach calculates metabolite fluxes based on the assumption that the system is at steady-state; therefore, intracellular metabolite production and consumption are balanced over relevant time intervals.

Flux balance analysis (FBA) is one type of CBM that uses linear optimization to identify solutions of metabolic models, based on an objective function which often involves maximizing the flux through a biomass synthesis reaction^13^. The FBA solution is a flux distribution that predicts cellular phenotype including which enzymes are active and what the magnitude of the flux is for each enzyme. The biomass synthesis reaction accounts for cell growth as observed experimentally^14^. FBA and derived methods are used to make in silico phenotype predictions based on changes in the growth medium or based on altering of enzyme activity through gene knockouts or recombinant interventions^15^.

Elementary Flux Mode (EFM) analysis is another CBM method that performs an exhaustive enumeration of the edges of the metabolic solution space defined by the stoichiometric matrix; FBA solutions are nonnegative linear combinations of EFMs^16^. The number of EFMs grows exponentially in relation to the number of reactions; counting all EFMs has been proven to be #P-hard^17^. Consequently, enumeration of EFMs from large metabolic models with over 100 reactions is challenging, requiring special computational methods^18^, biological constraints such as transcriptional regulation^19^ and thermodynamic data^20^, and careful model network compression^21,22^.

Building from the set of metabolic reactions encoded in the genome, and progressing to the intricate mechanisms at the protein and enzyme level, CBM contributes to the description of a wide variety of cellular processes. Genome-scale metabolic models (GSMMs), large-scale constraint-based metabolic models computationally generated from genomes of interest are now the norm^23,24^, due to increased availability of data and computational power. GSMMs are well suited for identifying putative drug targets through predicting gene and metabolite essentiality^25,26^.

GSMMs have been applied to analyse drug targets in cancerous cells^27,28^ and to treat *P. aeruginosa* infections^29^. The methods identified essential reactions and synthetic lethals (SLs)^30^. Synthetic lethals refers to combinations of gene-deletions or enzyme interference targets which prevent growth. While the term initially referred to pairs of genes, it is now used to describe n-tuples of reaction targets. The synthetic lethals may explicitly consider both the metabolic potential of the organism and the role of the nutritional environment provided by the extracellular medium.

Improved algorithms for computing synthetic lethal strategies have been proposed to shorten the calculation process, such as Fast-SL^31^ and Rapid-SL^32^. The computation of synthetic lethals deals with a combinatorial exploration of every possible n-tuple of reactions. Thus on large networks of over a thousand reactions, computation times increase as the size of n-tuples increase, and become impracticable for n-tuples of over 4 reactions^31^.

Another method proposed for identifying synthetic lethals, whether n-tuple size is under or over 4, is the computation of Minimal Cut Sets (MCSs), with the biomass synthesis reaction as a target reaction. MCSs are traditionally defined as the ’Minimal Hitting Sets’ of Elementary Flux Modes (EFMs)^17,33^, and are an exhaustive way of exploring robustness of a network. Setting a certain reaction as target for inactivation, MCSs define all sets of reactions capable of preventing flux through the target reaction^34^. MCSs analysis has demonstrated remarkable performance in identifying synthetic lethals in cancer cells^28^. Additionally, Minimal Cut Sets have been formalized for metabolic engineering and recombinant strain optimization^33^.

MCSs suffer the same computational time challenges as EFMs. The number of possible MCSs grows exponentially with the number of reactions^35^. Interestingly, it has been proven that MCSs can be enumerated as the EFMs of a so-called dual metabolic network^36^. As a result, similarly to how Mixed-Integer Linear Programming (MILP) methods were developed for computing the shortest EFMs of a metabolic network^37^, MILP methods for computing the shortest MCSs have been developed^38^.

Furthermore, it might be useful to convert the obtained MCSs into sets of target genes or proteins for biological interpretation. Gene-Protein-Reaction association rules (GPRs) have been developed for GSMMs and integrated into the stoichiometric matrix^39^. This data has been repurposed for the MCSs computation^28,40^, as a way to get intervention targets.

Here, we present a new approach for calculating and analysing MCSs using our *aspefm* tool^41^. The *aspefm* program is a logic programming method designed to compute subsets of EFMs while respecting user-defined constraints. It differs from the Double Description method, implemented in *efmtool*^42^, which needs to enumerate all solutions before generating results, and from MILP-based methods, implemented in *CNApy*^40,43^ and *CoBAMP*^44^, which minimize the size of the reaction set. We have extended the functionality of *aspefm* to the computation of MCSs.

Throughout this work, we distinguish MCSs of small size (reaction n-tuples of size 3 or less including essential reactions, synthetic lethal pairs and synthetic lethal triplets) from MCSs of large size (defined here as reaction n-tuples of size 4 or more). The MCSs of small size are often the desired reaction sets, they are readily calculated by MILP methods and SLs computation algorithms, and easily converted to gene and enzyme targets using GPRs. On the other hand, the MCSs of large size are less well-studied. While they are informative of network robustness, they might be harder to compute and interpret without incorporation of biological constraints. In this study, we show *aspefm* was able to bridge the gap between the two types of MCSs, using large-size MCSs to reveal possible interspecies metabolite interactions.

*aspefm* successfully processed an aggregate model of two GSMMs totalling over three thousand reactions: a consortium model of bacteria *S. aureus* and *P. aeruginosa*. The tool efficiently identified solutions of interest in acceptable computation times despite the size of the consortium metabolic network. The *aspefm* application revealed potential, nonobvious, interspecies metabolite exchanges that could help the consortium growth. Promising therapeutic targets for controlling the problematic pathogens were obtained by restricting the search of enzyme targets to knock-outs that were not nullified by consortial metabolite exchanges.

## Results

### Overview of genome-scale metabolic models for analysis of single species and consortium

Manually curated genome-scale metabolic models (GSMM) of *Staphylococcus aureus* and *Pseudomonas aeruginosa* were selected for our analysis. The *S. aureus* GSMM, *iYS854*, was developed based on *S. aureus* str. JE2^45^. The GSMM has been used for assessing the validity of experimentally determined transcriptional regulation modulons of *S. aureus*^46^. The model has been graded as the most accurate *S. aureus* GSMM currently available, according to a study by Renz and Dräger^47^.

The *P. aeruginosa* GSMM *iPae1146* is based on *P. aeruginosa* strain PAO1^48^. The GSMM was used for a high-throughput essentiality analysis^29^. In that study, the model was predicted to have around 97% accuracy for at predicting gene essentiality during growth on Lysogeny Broth (LB) medium.

Both metabolic models were pre-processed and curated for our analysis, as detailed in the Methods. The resulting *iYS854* model includes 1454 reactions, 1338 metabolites, and 866 genes, while the resulting *iPae1146* model includes 1495 reactions, 1283 metabolites, and 1148 genes.

The models were analysed in an in silico extracellular environment defined by CSP chemically defined medium^49^, on which *S. aureus* and *P. aeruginosa* can grow as biofilms in vitro^50^. The CSP medium was designed to serve as a simplified analogue of chronic wound exudate. The medium was chosen as the base for all predictions of growth and consortial cross-feeding in our study.

A consortium model consisting of *P. aeruginosa* and *S. aureus* was built from *iPae1146* and *iYS854* by adding metabolite exchange reactions with a shared control volume containing the growth medium. The newly created metabolic network contains 3241 reactions and 2752 metabolites. When the consortium model was constrained by an extracellular environment defined by CSP medium, a total of 57 metabolites were classified as ’external’ substrates. 46 external substrates were available to both *S. aureus* and *P. aeruginosa*, one metabolite was exclusively available to *P. aeruginosa*: citrate and ten metabolites were exclusively available to *S. aureus* including some vitamins and purines. A summary of medium metabolites is provided in Supplementary Table 2.

The network compression process, which is required for MCSs computation, excluded 1296 blocked reactions from the CSP-constrained consortium model and returned a compressed consortium network of 1062 reactions and 600 metabolites. Statistics and results from the construction and compression of the consortium model, and of the individual species models *iYS854* and *iPae1146*, are reported in Table 1.

**Table 1:** *Pseudomonas aeruginosa*, *Staphylococcus aureus* and consortium model statistics

Metabolic models	P. aeruginosa	S. aureus	Consortium
Model statistics			
Number of reactions	1495	1454	3241
Number of metabolites	1283	1338	2752
Number of genes	1148	866	2014
Number of external metabolites and exchange reactions	171	239	293
Number of reactions useable for cross-feeding	/	/	410 (239 + 171)
MEMOTE and consistency analysis results			
Universally blocked reactions	637	293	1031
Orphan metabolites (consumed but not produced)	54	39	93
Dead-end metabolites (produced but not consumed)	84	61	145
Reactions in stoichiometrically balanced cycles	253	46	697
Extracellular metabolites without exchange reactions	29	38	/
Compression results on CSP medium			
CSP medium metabolites	47	56	57
Blocked reactions on CSP medium	673	485	1296
Number of compressed reactions	470	505	1062
Number of metabolites of the compressed network	252	440	600
Consortium partner metabolites on CSP medium			
Number of metabolites secreted within the consortium model	65	73	/
Number of metabolites taken up within the consortium model	48	68	/
Number of metabolites taken up secreted by the other bacterium	36	47	/
Minimal cut sets of size three or fewer			
Essential reactions on CSP medium	183	309	2
Synthetic lethal pairs of reactions	184	282	over 50 ⋅ 10^3^
Synthetic lethal triplets of reactions	216	347	over 50 ⋅ 10^3^
Total number of MCSs of size three or fewer	583	938	unknown
After tests for consortium growth recovery			
Single-species MCSs nullified by consortium metabolite exchanges	68	199	/
Single-species MCSs remaining lethal on the consortium	515	739	/
Consortium-level MCSs with enzyme target potential	65		

Models were individually curated and compressed, then essential reactions, synthetic lethal pairs, and synthetic lethals triplets were computed on the single-species models and on the dual species consortium model. MCSs computations of synthetic lethal pairs and triplets on the consortium model were too expansive and thus stopped after one week. MCSs are given at the reaction-level on the uncompressed network.

As well, the numbers of MCSs of small size for the consortium and individual species models are reported in Table 1. 583 MCSs of size three or less were found for the single-species *iPae1146* model; 938 MCSs for the single-species *iYS854* model. Two single reactions are essential to the consortium-level model, the uptake of ferrous ions and the secretion of glycolate. These two conditions are necessary for the biomass synthesis reactions of both models.

**Fig. 1:**
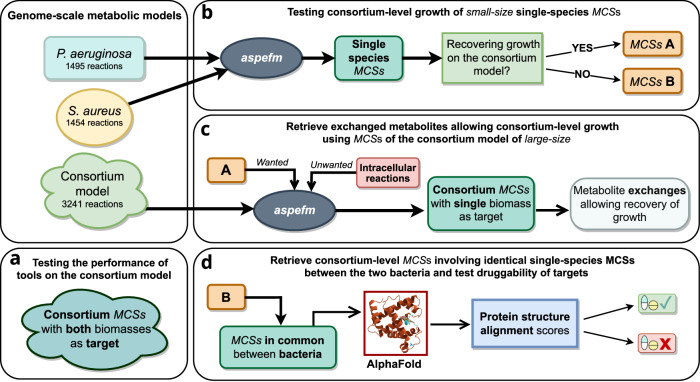
Overview of the main study. MCSs of size three or less are denoted as small-size MCSs. **a** Performance of MCSs computation tools on the *S. aureus* and *P. aeruginosa* consortium model is tested with consortium-lethal MCSs. **b** Small-size MCSs are computed on single-species models, then tested for growth recovery on the consortium-level model. **c** For MCSs which are no longer lethal on the consortium model, larger size consortium MCSs are computed, revealing metabolite exchanges. **d** For MCSs that are still lethal on the consortium retrieved in both bacteria, interspecies protein structure alignments of AlphaFold structures are performed, and the best drug targets for therapeutic intervention in human are retrieved.

As per Fig. 1, our study relies on small-size single-species MCSs as queries to derive larger-size consortium-level MCSs. While small-size MCSs could be computed using existing MCSs tools or the Synthetic Lethals algorithms, there are differences in computational performance between *aspefm* and the other MCSs computation tools as summarized in Supplementary Table 1. These differences motivate the continued study of MCS algorithms to identify best practices for specific scientific inquiries.

Single-species MCSs are classified based on their ability to rescue growth in the consortium model. In a prelimlinary step, performance of *aspefm* was compared to MCSs tools *CNApy*^43^ and *CoBAMP*^44^ for computation of large-size solutions on the consortium model. In a second step, single-species MCSs that rescued growth were used to derive the exact metabolite exchanges that nullified the cut set from the consortium model through a constrained consortium MCSs analysis. As a final step, single-species MCSs that did not rescue growth were used to develop a ranking of consortium-level enzyme targets based on protein structure similarity.

For the final step, single-species MCSs that did not rescue growth and were identical in both microorganisms were collected and gathered into larger reaction sets. These reaction sets are, by definition, consortium-level cut sets. This approach was deemed better than a constrained MCSs analysis on the consortium model for two reasons. First, MCSs tools could not perform unconstrained enumeration of small-size MCSs of the consortium model in reasonable time and second, MCSs at the consortium-level mostly consisted of combinations of single-species MCSs. Enzyme targets were derived from cut sets that could correspond to single enzyme pairs.

### *aspefm* efficiently calculates MCSs from consortium-level models regardless of the size of the reaction set

The performance of our *aspefm* tool was evaluated by computing MCSs from the compressed consortium model comprised of 1062 reactions. The target reactions for the simulation were the biomass synthesis reactions for both single species. The only constraints for the simulation were to identify MCSs of reaction size 16 or less and a maximum run time of 1.5 days.

**Fig. 2:**
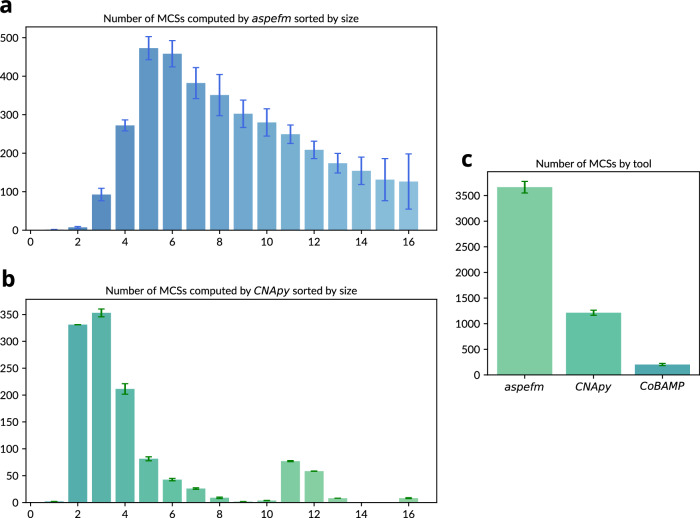
Number of compressed network MCSs computed from the consortium model by each tool with a time limit of 1.5 days, and limited to MCSs of below 16 reactions. **a** Size of MCSs computed by *aspefm*, **b** Size of MCSs computed by *CNApy*, **c** Number of MCSs computed by each tool. Heights correspond to average numbers for five program executions, error bars represent standard deviation around the mean for figure (**c**), and half of standard deviation for (**a**) and (**b**).

The performance of *aspefm* was compared with *CNApy*^43^ and *CoBAMP*^44^, both are MILP-based MCSs enumeration methods. For each tool, five executions were launched and averaged (Fig. 2). On average, *aspefm* identified more than thrice as many compressed MCSs as *CNApy* in the 1.5 day run time. *aspefm* averaged 3663.8 MCSs while *CNApy* averaged 1213.0 MCSs. Both *aspefm* and *CNApy* were able to enumerate MCSs regardless of the reaction set size, while *CoBAMP* was heavily hindered by its forced iterative enumeration approach which started with the smallest cut sets; the method only identified around 203.6 MCSs on average. Upon decompression, the MCSs found by *aspefm* and *CNApy* reached the order of 10^5^ MCSs, illustrating the necessity of network compression.

The MCSs identified using *aspefm* sampled solutions ranging from 1 to 16 reactions with the highest frequency at a reaction size of 5 (Fig. 2). As illustrated by the high variability over 5 executions, no substantial preference was observed towards any particular reaction size: *aspefm* is a logic programming tool that benefits from the combinatorial exploration nature of its SAT-based capabilities. Meanwhile, the MCSs identified using *CNApy* were biased toward smaller reaction sizes and mainly enumerated solutions with 2–7 reactions, with the highest frequency occurring at 3 reactions (Fig. 2). These results from the consortium model suggest that in contrast to *CNApy* and especially *CoBAMP*, *aspefm* enumerated solutions independently of their size. For computing MCSs of large size from the consortium model, *aspefm* is thus the tool of choice.

### Consortium-level MCSs reveal role of interspecies metabolite exchanges in single-species growth

Medical infections comprised of both *S. aureus* and *P. aeruginosa* can result in worse patient outcomes and can be more difficult to treat than monocultures. Thus therapeutic treatments ideally should not only target a single species, but the whole consortium. Metabolic modelling can identify metabolite exchanges between species that would bypass therapeutic strategies targeting only a single species. MCSs of small size from the single-species models were tested for lethality at the consortium level to determine if directed cross-feeding interactions or passive metabolite exchange through metabolite leaking could circumvent single-species lethalities. Of the 583 MCSs of size three or less for *P. aeruginosa*, 68 were no longer cut sets at the consortium level based on metabolites secreted by *S. aureus*. Meanwhile, of the 938 MCSs of small size for *S. aureus*, 199 cut sets were no longer effective due to metabolite exchanges from *P. aeruginosa*.

The lethal MCSs that were nullified due to metabolite exchanges were further analysed with the consortium-level model. *aspefm* was chosen for determining the identity of the exchanged metabolites, since the necessary computation involved consortium-level MCSs of large size: over three reactions for the most part. Constraints for the computation were separated into two categories. We defined within *aspefm*: ‘wanted reactions’ (positive Boolean inputs) for reactions that should appear in the MCSs, and ‘unwanted reactions’ (negative Boolean inputs) for reactions that should not appear in the MCSs; the latter should not be confused with the target reaction. *aspefm* was run on the consortium model for each nullified MCS and for each bacterium, the reactions from the invalidated MCS were set as the ‘wanted reactions` and all reactions unnecessary for metabolite exchange as the ‘unwanted reactions’; the target reaction was set to the single-species biomass reaction (see Methods). As a result, generated consortium-level MCSs were composed only of the previously computed single-species MCSs and of exchange reactions, revealing the metabolites essential for recovering growth.

The obtained MCSs were limited to eight or fewer reactions and a time limit was set to 1.5 days for each computation. In total, 531 compressed consortium model MCSs were computed, ranging in size from 2 to 8 reactions, with the mean and median being 6 reactions and the highest frequency being 7 reactions. The details of each consortium-level MCS identified through this analysis are provided in Supplementary Dataset 1.

**Fig. 3:**
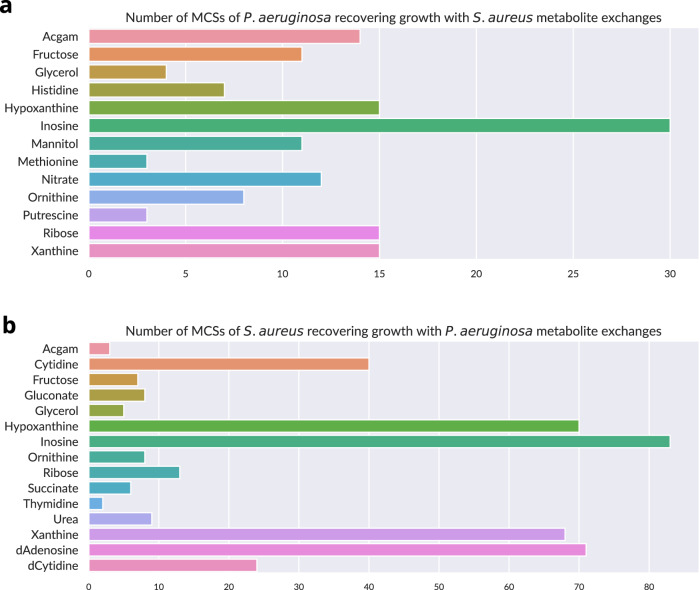
Metabolite exchanges with consortium partners allowing single-species MCSs to recover growth on the consortium model, according to consortium MCSs of size up to 8, found in a 1.5 days time span. **a** Number of MCSs of *P. aeruginosa* nullified by extracellular metabolite import from *S. aureus*, **b** Number of MCSs of *S. aureus* nullified by extracellular metabolite import from *P. aeruginosa*. Acgam acetylglucosamine, d deoxy.

The identity of the exchanged metabolites in the consortium model and the number of single-species MCSs they suppress are reported for each bacterium in Fig. 3. The same single-species MCSs could be invalidated by the exchange of any one of several metabolites. Indeed, for a given single-species MCS, several consortium MCSs could be found, indicative of intervention strategies at the consortium level, and corresponding each to one or several metabolites.

For instance, a MCS of size three that exists for a single-species model might be nullified by any of five different metabolite exchange reactions, together in a consortium model MCS of size eight. Consequently, at least five interventions on exchanges with the other bacterium would be required for the original MCS to regain lethality. Alternatively, if among all consortium MCSs, there was only one metabolite exchanged to counter the single-species MCS, then only a single theoretical intervention would be required.

The majority of cut sets nullified due to metabolite exchanges involved purine metabolism, pentose phosphate pathway, and glycolysis. Inosine was a pivotal metabolite in many of those functions, it was able to complement almost half of the identified cut sets for each bacterium. Inosine nucleosidase can transform the metabolite into hypoxanthine—a purine – and ribose, which can support many central metabolism transformations.

Purines xanthine and hypoxanthine seemed to play a central role in metabolite exchanges allowing the recovery of growth, likely due to their relationship with nucleotide metabolism. Other notable metabolite exchanges shared by the two bacteria include acetylglucosamine, ribose, fructose and glycerol, complementing glycolysis functions; and urea-related metabolites, complementing urea cycle metabolism functions.

Previously, the enzyme N5,N10-methylenetetrahydrofolate dehydrogenase-cyclohydrolase, catalysing methylenetetrahydrofolate dehydrogenase and methenyltetrahydrofolate cyclohydrolase, has been studied as potential drug target for *P. aeruginosa*^51^. However, these reactions, identified as essential on the single-species model, are complemented by purines and histidine exchanged by *S. aureus* on the consortium-level model.

Although the majority of the single-species level MCSs corresponded to large-size consortium-level MCSs: most recovering growth by at least two distinct possible metabolite exchanges and making for very impractical treatments, we found a few small-sized MCSs, such as glucosamine-6-phosphate synthase with acetylglucosamine exchange.

Interestingly, while glucosamine-6-phosphate synthase was identified as an essential reaction in both single-species models, the lethal phenotype conferred by deleting this reaction would be recovered by a simple exchange of acetylglucosamine between the bacteria. Therefore, efforts using therapeutic agents which inhibit this enzyme^52^ would need to consider the potential role of acetylglucosamine found in the environment or leaked by resistant bacteria in the consortia.

### Consortium-level MCSs identify multi-species, structurally close, intervention targets

The effects of therapeutic agents that target a single species can, in some cases, be bypassed through metabolite exchange from other consortia species. From single-species MCSs, our analysis illustrated how specific consortium-level metabolite leakage or cross-feeding events could enable growth recovery. This identified MCSs that should be excluded from further analysis, while the remaining MCSs could be more promising targets for therapeutic intervention.

In order to retrieve consortium-level intervention targets, we selected MCSs that were mutually shared by the two bacteria. As well, the corresponding enzyme targets were retrieved from MCSs for protein structure studies. It is hypothesized that proteins with similar structure could be targeted simultaneously by the same therapeutic agent and thus target the consortium by conjointly blocking both *S. aureus* and *P. aeruginosa*. Out of 515 *iPae1146* MCSs and 739 *iYS854* MCSs of small size that were not nullified by metabolite exchanges, 65 MCSs were shared by both bacteria (Table 1).

**Fig. 4:**
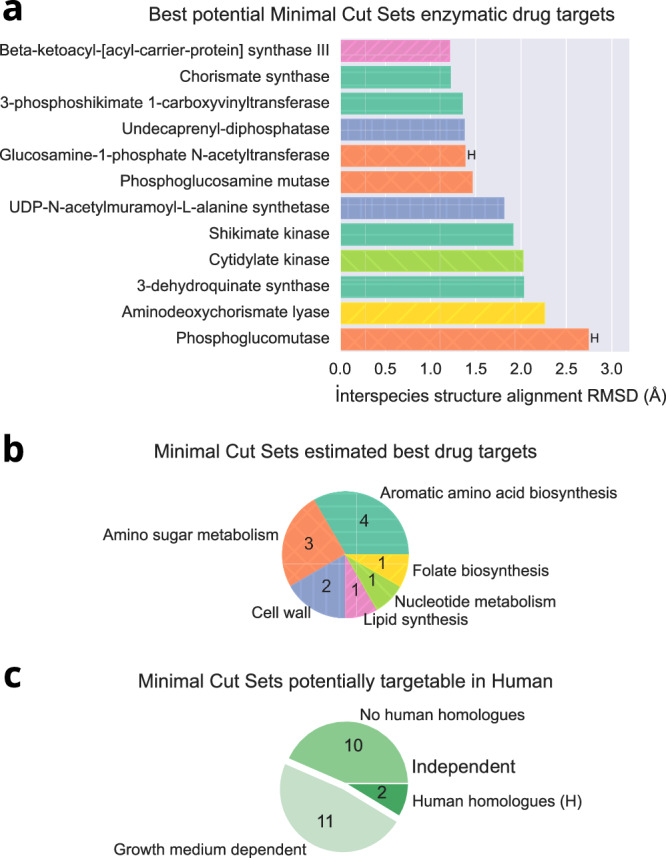
Consortium MCSs estimated to be targetable with a single ligand. Only *P. aeruginosa* and *S. aureus* targets whose protein structures matched best were kept. **a** Ordinates labels show targetable enzymes. Bars heights are Root Mean Square Deviation (RMSD) of atom positions, measured in Ångström, resulting from protein structural alignment of enzyme pairs. Enzymes indicated with ‘**H**’ were found to have human homologues. **b** Pie distribution for associated metabolism groups for each targetable enzyme in **a**. **c** Pie distribution of categories of MCSs potentially targetable in human. From the 23 MCSs targetable in human (in **c**), only 12 are independent of the growth medium (shown in **a** and **b**).

The structures of the proteins associated with the shared 65 MCSs were analysed for similarity. The protein structure analyses ruled out several MCSs since the target enzymes for *S. aureus* and *P. aeruginosa* were not deemed similar enough for concurrent therapeutic intervention. Enzymes were represented by their GSMMs gene assignments, ie. GPRs (Gene-Protein-Reaction gene products), and were retrieved from MCSs with a logic programming extension of our *aspefm* procedure. Interspecies relative enzyme structure similarity was measured by Root Mean Square Deviation (RMSD) of atom positions in Ångström, protein structure alignments were computed on AlphaFold structure predictions^53^. The remaining shared MCSs were filtered using an additional criterion: the *S. aureus* and *P. aeruginosa* protein structures were compared to any potential human homologues. MCSs with enzymes that were deemed too similar to human homologues were removed. As a result, a ranking of the most promising MCSs for antibiotics discovery in human is presented in Fig. 4.

From the 65 MCSs common to both bacteria and their corresponding enzymes, 23 of them were considered good potential drug targets for elimination of the bacterial consortium in human (Fig. 4c). Details and ranking of each MCS are provided in the Supplementary Dataset 2. The most promising enzymes to simultaneously target both *S. aureus* and *P. aeruginosa* included twelve enzymes from nucleotide metabolism, lipid synthesis, aromatic amino acid biosynthesis, bacterial cell wall construction, amino sugar metabolism and folate biosynthesis (Fig. 4a, b).

At the top of the ranking, beta-ketoacyl-ACP synthase III had high protein structure similarity between the two bacteria (interspecies RMSD: 1.21 Å) and fortuitously, there did not seem to be human homologues. However, there exist functionally related isozymes beta-ketoacyl-ACP synthase I and II, presenting high similarity with human homologues, which were thus excluded by our procedure.

Some example cell wall synthesis enzymes include undecaprenyl-disphosphatase (interspecies RMSD: 1.37) and UDP-N-acetylmuramoyl-L-alanine synthetase (RMSD: 1.81). Both enzymes are found only in the bacteria and not in human. Amino sugar metabolism included two enzymes for which there are human homologues: GlmU and phosphoglucomutase, however the protein structures were considered dissimilar enough by our filtering procedure for these enzymes to be considered targetable.

Other enzymes with high therapeutic potential to treat *S. aureus* and *P. aeruginosa* consortia are four enzymes from the aromatic amino acid biosynthesis pathway: chorismate synthase (RMSD: 1.22), 3-phosphoshikimate 1-carboxyvinyltransferase (RMSD: 1.35), shikimate kinase (RMSD: 1.91) and 3-dehydroquinate synthase (RMSD: 2.03). This biosynthesis pathway is famously not present in mammals. In fact, an inhibitor of 3-phosphoshikimate 1-carboxyvinyltransferase, glyphosate, is commonly used as an herbicide^54^.

Additionally, eleven environment-dependent, bacterial-only enzyme targets were identified (Fig. 4c). These enzymes are derived from 2–3 reaction MCSs containing a transporter for an amino acid found in the growth medium and an amino acid biosynthesis reaction, which becomes essential in absence of that amino acid. Were the bacteria to be grown in an environment lacking the amino acids associated with these MCSs, an inhibitor of the enzyme targets would be effective.

These MCSs provide detailed, systemic insight into which amino acid biosynthesis reactions are the most important for *S. aureus* and *P. aeruginosa* growth, and therefore which amino acids biosynthesis pathways are the most promising targets. Our eleven MCSs correspond to six important amino acids, among two classes, aromatic amino acids (tryptophan, histidine, phenylalanine) and branched-chain amino acids (isoleucine, leucine, valine). These essential pathways are in accordance with previous studies and might be druggable^55^. More insight into the growth medium dependent MCSs, as well as structure alignments and human homologues, is given in Supplementary Material, Supplementary Figs. 2 and 3.

## Discussion

*aspefm* is a powerful and promising tool for metabolic systems analysis, able to compute EFMs while respecting any applied constraints, whether they are logical or linear, and it can be extended at will by the *clingo* Python interface^56^. Here, we extend the tool to the computation of MCSs from metabolic models of single and multispecies systems. MCSs are mathematical objects with great engineering, ecological, and therapeutic potential as they identify combinations of reactions within large, highly connected networks that have outsized abilities to influence phenotype. As illustrated in Fig. 2, exploration of MCS solutions in genome-scale models is now possible. Compared to other methods, *aspefm* showed substantially better performance for enumeration of MCSs of large size on the consortium model within a time frame of 1.5 days. Note that the enumeration of these subsets of solutions with *aspefm* is non-ordered and non-deterministic, as demonstrated by the variability in MCSs sizes. In contrast, *CoBAMP*’s enumeration algorithm is ordered, consisting of computing the next smallest MCSs at each iteration. *CNApy* proposes both ordered and non-ordered enumeration, *aspefm* was compared to its non-ordered enumeration. With its innovative SAT-based solving, our tool *aspefm* was able to enumerate solutions on a consortium-level with ease, despite the network comprising about three thousand reactions.

In order to make full use of the enumeration capacities of our *aspefm* tool, we devised a MCSs analysis to study potential cross-feeding interactions within the consortium. Our analysis proposed the usage of MCSs of small size on single-species models as constraints for the computation of MCSs of large size on a consortium-level (Fig. 1). Single-species MCSs were tested for the recovery of growth on the consortium model, and if metabolite exchanges permitted growth recovery, then MCSs of large size were computed, providing insight into the metabolite exchanges in question (Fig. 3). To do so, all reactions that were not metabolite exchange reactions were set as negative Boolean inputs, and MCSs of small size were used as positive Boolean inputs, and *aspefm* was run with a time limit of 1.5 days. We implemented an additional FBA check for all MCSs identified by the solver to verify the minimality of the solutions. Therefore we present the utility of an exhaustive yet constrained metabolic pathways analysis, through application of biological relevant constraints, as we have presented previously for EFM analysis^41^. Rather than a complete enumeration of all cut sets, which like EFM enumeration is only achievable on modestly sized reaction networks, we enumerate a subset of MCSs while answering a specific biological question, under a finite time limit.

This new application of MCSs highlights the potential role of metabolite exchanges and metabolite cross-feeding on consortium functioning and resilience to therapeutic efforts (Fig. 3). We strengthened the study by combining the analysis with drug target predictions for the single-species MCSs that did not regain growth with consortial metabolite exchanges (Fig. 4). Meylan and coauthors have showed strong evidence that antibiotic tolerance might be affected by the impact of metabolite exchanges, in particular they showed that uptake of fumarate/glyoxylate by *P. aeruginosa* increases/decreases respectively its tolerance to aminoglycosides^57,58^. Usually, MCSs of size three or fewer are considered the most biological relevant. This logic follows the argument that disrupting more than three different genes, or interfering with more than three mRNA targets or the drugging of more than three enzymes at the same time is challenging. MCSs of small size are readily enumerated from single-species metabolic models, and can be performed with existing SLs enumeration tools or with MCS enumeration tools. However, we argue that MCSs of large size can provide valuable information on network robustness and fragility. MCSs of large size cannot be reliably enumerated by SLs tools as these are iterative methods not adapted for combinatorial enumeration. Here, MCSs of large size revealed the explicit metabolite exchanges between the two bacteria that enabled recovery of growth after three or fewer reactions were cut. We believe this methodology can be extended to larger aggregate models including microbiome-level systems and be used to systematically identify metabolite exchanges that can and can not circumvent medical treatments.

We validated the our predicted metabolite exchanges uncovered with MCSs using COBRAPy and FBA to test all possible metabolite cross-feeding interactions; the results are reported in Supplementary Table 3. Of note, metabolite exchanges can also be retrieved on bacterial consortia models through using EFMs analysis^59,60^. Interestingly, we compared our tool to SMETANA, a tool for estimating growth-dependent species exchanges in bacterial consortia^61^. 25% of our highlighted metabolite exchanges were not predicted by SMETANA. The analysis performed by SMETANA on our consortium model is given in Supplementary Fig. 4. We believe interspecies metabolite exchanges are representative of at least three possible mechanisms: cross-feeding (bacterial co-operation), metabolite leakage (non specific loss of metabolites necessary for biomass production), and metabolite acquisition from the growth medium which may include necromass^62^.

Our study positions itself in recent efforts from the metabolic modelling community in bringing metabolic models to the cellular consortium level^63^. There is a growing interest in multi-species models such as AGORA for modelling of gut microbiota^64^, and multicellular models such as whole-body human models^65^. However, we found that even the well-curated single-species models presented in our study were not exempt from curation issues. For example, *iPae1146* lacked reactions for aminoacyltransferase reactions. Interestingly, the aminoacyltransferases reactions were present on the smaller *P. aeruginosa* PAO1 model *iMO1056* from 2008^66^. Another curation issue included the lack of a functioning formate transporter for *iPae1146* and of a citrate transporter for *iYS854*, even though these metabolites might have substantial interspecies exchange potential.

Data from UniProt and AlphaFold^53^ was used to predict therapeutic targets based on protein structure similarities. Protein structure data was not available for many of the enzymes of interest which limited the mapping of the specific protein sequence to a model structure. We therefore used the application to quantify similarities of the predicted structure of two protein sequences rather than the overall accuracy of the 3D structure. If the predicted protein structures were deemed similar enough, it is hypothesized that an inhibitor would be more likely to be a ligand to both. AlphaFold derived preemptive ranking for further analysis and provided a uniform method of applying the computational workflow. Enhanced predictions will be possible with improved crystallization-based protein structures, and through further analysis of the enzyme targets, their active sites, and medically approved inhibitor ligands^67^.

Finally, a relevant point of discussion is the conversion of enzymes to gene and reaction data, ie. Gene-Protein-Reaction rules (GPRs). GPRs are composed of AND and OR Boolean rules, symbolizing complexes and isozymes respectively; GPRs are associated to the reactions they catalyse. Previous studies have incorporated GPRs into the stoichiometric matrix^39,40^. This has the downside of forcing flux through artificial reaction constructs. In contrast, our approach for converting reactions to proteins using GPRs made use of logic programming, – as GPRs are Boolean logic rules – thus further expanding the *aspefm* framework. GPRs are of major importance when analysing the enzymes gene or protein data, and were useful for us when retrieving AlphaFold entries^53^.

To conclude this study, we argue that biomass-associated MCSs of small size and of large size were equally useful. With our method, a subset of all large sized MCSs was able to reveal consortium-level metabolite exchanges that could only be observed after analysis of small sized MCSs. Additionally, MCSs which could not be complemented based on interspecies metabolite exchanges were analysed for their role as drug targets. We thus propose that there is a strong need for MCSs enumeration tools such as *aspefm*, and for metabolic modelling methods as a whole in the context of microbial ecology, medical intervention, and drug discovery.

## Methods

Minimal Cut Sets were computed on *Staphylococcus aureus* and *Pseudomonas aeruginosa* GSMMs *iYS854* and *iPae1146*, as well as on a consortium model containing the two models and reactions to model cross-feeding. The tools used are *aspefm*, our tool, for which we detail the methods further, *CNApy* and *CoBAMP*. For representing *CNApy*, the *StrainDesign* Python module was chosen and configured with the non-ordered enumeration algorithm^68^. We provided code for the analysis at https://github.com/maxm4/paSAmcs/. The repository includes the models and scripts for running every tool. We used IBM © *cplex* for linear programming solving and an Intel® Xeon® E5-2609v2 2.5GHz processor.

An overview of the complete analyses performed in Table 1, Figs. 3 and 4 is found in Fig. 1. MCSs of small size of *P. aeruginosa* and *S. aureus* were tested for growth recovery on the consortium model. For MCSs which lost their lethal phenotype on the consortium, MCSs of large size were retrieved explaining which metabolite exchanges allow recovery of growth. For the remaining MCSs of small size, MCSs in common between the bacteria were retrieved and analysed for the search of possible new antibacterial agents. This analysis is further illustrated in Supplementary Material: Supplementary Figs. 1, 5, and 6.

### Metabolic models pre-processing and curating

Both models went into a first phase of pre-processing. Erroneous identifiers were resolved and renamed. A pyocyanin transporter was added to *iPae1146*, as *P. aeruginosa* is known to secrete pyocyanin in presence of *S. aureus*^10^. As well, new transporter reactions were added to *iYS854* to match the Chemically Defined Medium used in ref. ^69^, and such that the well-studied WTA-null non-lethal *Staphylococcus aureus* mutant Δ *tarO* could be accounted for, in order to resolve the ambiguities raised by ref. ^45^.

Accuracy of the models was estimated using the MEMOTE community tool for assessing GSMM quality^70^. *iPae1146* scored low at 23 %, mainly due to its lack of annotations, while *iYS854* scored at 75%. In addition, network topology issues were reported in Table 1. The *iPae1146* model contains a smaller number of exchange reactions, about twice as many blocked reactions, and a substantially higher number of reactions implicated in stoichiometrically balanced cycles.

As well, 29 extracellular metabolites from *iPae1146* and 38 from *iYS854* were found to lack exchange reactions. Notable metabolites from these lists, which are thus excluded from the possible metabolic interactions between bacteria, include formate for *iPae1146* and citrate for *iYS854*.

The models were constrained to CSP Chemically Defined Medium^50^. For all 47 CSP medium metabolites metabolized by *P. aeruginosa* and all 56 CSP medium metabolites metabolized by *S. aureus*, exchanges lower flux bounds were set to flux values in accordance with their relative quantity in the medium. The obtained constrained metabolic models are presented in Supplementary Dataset 3, in the standard SBML format.

### Consortium model construction and analysis

**Fig. 5:**
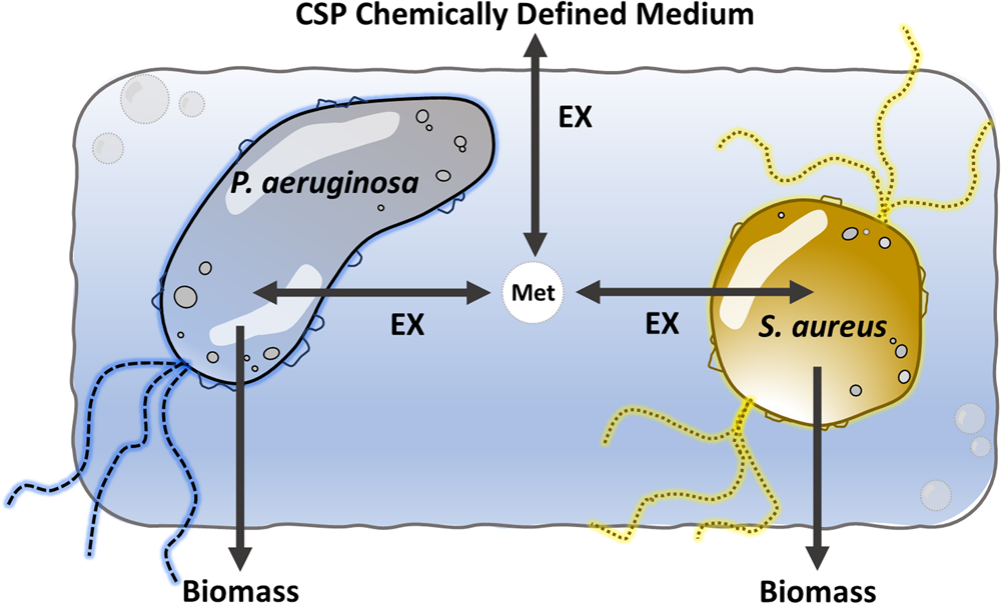
Diagram of the *P. aeruginosa* and *S. aureus* consortium model in CSP Chemically Defined Medium, including a view of metabolite exchange mechanisms. Extracellular metabolites are symbolized by “Met”. Exchange reactions are symbolized by “EX”.

A consortium model of *P. aeruginosa* and *S. aureus* models was constructed using exchange reactions of both models as means for cross-feeding. All reactions, metabolites and compartments of the original models were subtitled with ‘PA’ or ‘SA’ in the consortium model depending their origin. The consortium model’s highest level compartment permited exchange of metabolites between *P. aeruginosa* and *S. aureus*, as illustrated in Fig. 5.

Additional boundary exchange reactions were added to all newly created extracellular metabolites using COBRAPy^71^, and the new exchange reactions fluxes were constrained to correspond to metabolites of the CSP Chemically Defined Medium^50^. All reactions that were previously exchange reactions of the *iPae1146* and *iYS854* GSMMs have become reactions that can be used for cross-feeding. We set arbitrary flux bounds of [−20, 20] for all cross-feeding reactions, in accordance with the minimum possible uptake flux in CSP Medium, which is set to −20 mmol/gDW/hr for O_2_, as is standard for that metabolite in COBRA models. The resulting consortium metabolic model in SBML is presented in Supplementary Dataset 3.

Supplementary Table 3 presents the possible metabolite exchanges between the bacteria, as identified with COBRAPy tests. Within the consortium model, *P. aeruginosa* can metabolize 36 metabolites of the 73 metabolites potentially secreted by *S. aureus*, while *S. aureus* can metabolize 47 metabolites of 65 metabolites potentially secreted by *P. aeruginosa*. These metabolites are reported in Table 1 as well.

Note that for computation of MCSs—and thus essential reactions, synthetic lethal pairs and triplets—on a model alone, its biomass is taken as the target reaction. As such, when looking for which cross-feeding reactions complement a cut set of *P. aeruginosa* or *S. aureus* alone (Fig. 3), only one of the two consortium biomass reactions should be taken, the targeted biomass reaction in question. The procedure for computing MCSs revealing cross-feeding interactions with *aspefm*, from the sets of lethal MCSs with growth recovery of either bacterium, is illustrated in Supplementary Fig. 6.

Separately, for modelling growth on the consortium model, the FBA objective reaction is defined as the sum of both biomass reactions. Thus, for computation of MCSs which are lethal to the whole consortium model (Fig. 2), both biomasses were taken as the target reactions.

### Network compression

To help with computation efficiency, models are compressed using the network compression procedure developed by the von Kamp team, as part of the *pip* package efmtool_link from the Klamt lab (https://github.com/cnapy-org/efmtool_link). The tool relies on excluding blocked reactions and correcting reversibilities through Flux Variability Analysis^13^, then applying a nullspace-based compression method from *EFMTool*^42^.

The general principle behind nullspace-based compression was introduced in *METATOOL*^21^, and later re-explored in ref. ^22^. Linearly dependent lines from the stoichiometric kernel are regrouped into a single (lumped) reaction. This allows reactions that always operate together, i.e., their fluxes are linearly dependent to each other, to be regrouped into the same reaction subsets. For MCSs analysis, the linear coefficient factor between reactions in the same subset is of no importance, thus MCSs decompression is trivial.

### Network dualization

As described in Ballerstein et al.^36^, it is possible to describe the problem of computing MCSs as the problem of computing particular EFMs on a dual metabolic network, meaning that the original network has to undergo a dualization conversion procedure.

We formulated the problem by making use of the MILP version proposed by von Kamp and Klamt in 2014^38^, which excludes some of the linear variables and constraints introduced by Ballerstein. A notable feature of this method was defining an inequality constraint instead of an equality constraint for metabolites of the dual network that were originally irreversible reactions.

All reversible dual reactions are split into two irreversible dual reactions. As in the formalisms defined by Ballerstein and von Kamp, the reactions corresponding to reversibility constraints are the only ones to which subset-minimality applies, meaning the other linear variables are free to be either strictly positive or equal to zero following whether it suits the linear program. We provide Supplementary Fig. 7 to explain network dualization with *aspefm*.

### Dual metabolic network formalization

Let us define *S* the stoichiometry matrix of size *m* × *r*, *m* being the number of metabolites in the metabolites set ${{{\mathcal{M}}}}$ and *r* being the number of reactions in the reactions set ${{{\mathcal{R}}}}eac$. Let us define the set of reversible reactions ${{{\mathcal{R}}}}ev\subset {{{\mathcal{R}}}}eac$ and the set of target reactions $t\subset {{{\mathcal{R}}}}eac$ to be disabled for MCSs computation.

In the dual metabolic network, original reactions become metabolites, and original constraints become reactions. If *S* is the primal stoichiometry matrix is of size *m* × *r*, with **I** the identity matrix of size *r* × *r* and **−T** vector of size *r* × 1 with values 0 for *j* ∉ *t* and − 1 for *j* ∈ *t*, then ${{{\mathcal{D}}}}$ is a dual matrix of size *r* × *d*, with *d* = *m* + *r* + 1, and defined as ${{{\mathcal{D}}}}=({S}^{T}{{{\bf{I}}}}-{{{\bf{T}}}})$.

The computation of the dual network ${{{\mathcal{D}}}}$ generates a metabolites set ${{{{\mathcal{M}}}}}_{dual}$ and a reactions set ${{{\mathcal{R}}}}ea{c}_{dual}$. The set ${{{{\mathcal{M}}}}}_{dual}$ is simply the reactions set ${{{\mathcal{R}}}}eac$ and is thus of size *r*, while the reactions set ${{{\mathcal{R}}}}ea{c}_{dual}$ is of size *d* and composed of three different types of reactions: ${{{\mathcal{R}}}}ea{c}_{dual}={{{\mathcal{S}}}}\cup {{{\mathcal{RC}}}}\cup {{{\mathcal{T}}}}$.

More precisely, all *m* stoichiometry constraints ${{{\mathcal{S}}}}$ become reversible reactions, *r* reversibility constraints ${{{\mathcal{RC}}}}$ become reversible if and only if the original reaction is reversible too, while the reactions *t* to be disabled become one irreversible target reaction ${{{\mathcal{T}}}}$. More precisely, all *m* stoichiometry constraints ${{{\mathcal{S}}}}$ become reversible reactions, *r* reversibility constraints ${{{\mathcal{RC}}}}$ become reversible if and only if the original reaction is reversible too, while the reactions *t* to be disabled become one irreversible target reaction ${{{\mathcal{T}}}}$.

After splitting the *d* dual network reactions into *k* irreversible reactions, the set ${{{{\mathcal{R}}}}}_{dual}$ of irreversible reactions is obtained. For clarity, *k* = 2*m* + *r* + ∣*Rev*∣ + 1. For MCSs computation, we are concerned with its subset of interest ${{{\mathcal{C}}}}ut\subset {{{{\mathcal{R}}}}}_{dual}$, corresponding to the split of reactions ${{{\mathcal{RC}}}}$. The set $Re{v}_{dual}:{{{{\mathcal{R}}}}}_{dual}\times {{{{\mathcal{R}}}}}_{dual}\to {{{\mathcal{R}}}}ea{c}_{dual}$ keeps track of which reactions of ${{{{\mathcal{R}}}}}_{dual}$ were originally reversible in the reaction set ${{{\mathcal{R}}}}ea{c}_{dual}$. This is the set of forwards backwards reaction pairs. Further ahead, we denote by *D* the dual matrix of size *k* × *r* after splitting reversible reactions.

### Minimal Cut Sets formalization

Given ${{{\mathcal{R}}}}ev\subset {{{\mathcal{R}}}}eac$ the set of reversible reactions of the primal network, *D* the dual matrix of the stoichiometric matrix *S*, ${{{{\mathcal{M}}}}}_{dual}$ the set of dual metabolites, originally reactions of the primal network, ${{{{\mathcal{R}}}}}_{dual}$ the set of *k* irreversible dual reactions, $Re{v}_{dual}\subset {{{{\mathcal{R}}}}}_{dual}\times {{{{\mathcal{R}}}}}_{dual}$ indicating which pairs of reactions result from a split, ${{{\mathcal{C}}}}ut\subset {{{{\mathcal{R}}}}}_{dual}$ the subset of reactions corresponding to directionality constraints of the primal network, and ${{{\mathcal{T}}}}\in {{{{\mathcal{R}}}}}_{dual}$ a target reaction associated to one or several primal network reactions *t* that should be disabled, the Minimal Cut Sets problem can be defined as the following:

Problem: Find non trivial subset-minimal assignments to {*True*} of ${c}_{\overline{r}}\in {\mathbb{B}}$, $\forall \overline{r}\in {{{\mathcal{C}}}}ut$ such that :

1 $$\mathop{\sum}\limits_{\overline{r}\in {{{{\mathcal{R}}}}}_{dual}}{D}_{\overline{m}\overline{r}}\times {v}_{\overline{r}}=0\quad \forall \overline{m}\in {{{{\mathcal{M}}}}}_{dual}\cap {{{\mathcal{R}}}}ev$$

2 $$\mathop{\sum}\limits_{\overline{r}\in {{{{\mathcal{R}}}}}_{dual}}{D}_{\overline{m}\overline{r}}\times {v}_{\overline{r}}\ge 0\quad \forall \overline{m}\in {{{{\mathcal{M}}}}}_{dual}\setminus {{{\mathcal{R}}}}ev$$

3 $${v}_{\overline{r}}\ge 0\quad \forall \overline{r}\in {{{{\mathcal{R}}}}}_{dual}$$

4 $${z}_{\overline{r}}\iff {v}_{\overline{r}} > 0\quad \forall \overline{r}\in {{{{\mathcal{R}}}}}_{dual}$$

5 $${c}_{\overline{r}}\iff {z}_{\overline{r}}\quad \forall \overline{r}\in {{{\mathcal{C}}}}ut$$

6 $$\neg {z}_{\overline{r}}\vee \neg {z}_{{\overline{r}}_{rev}}\forall (\overline{r},{\overline{r}}_{rev})\in Re{v}_{dual}$$

7 $$v\in {{\mathbb{R}}}^{k},z\in {{\mathbb{B}}}^{k},{v}_{{{{\mathcal{T}}}}} > 0$$

${D}_{\overline{m}\overline{r}}$ denotes the dual matrix *D* stoichiometry coefficient associated to dual metabolite $\overline{m}\in {{{{\mathcal{M}}}}}_{dual}$ and dual reaction $\overline{r}\in {{{{\mathcal{R}}}}}_{dual}$. Equations (1) and (2) represent the steady-state constraint, and is an equality or an inequality whether the metabolite the constraint applies on was originally a reversible or an irreversible reaction. Equation (3) is defining that all reaction fluxes ${v}_{\overline{r}}$ should be positive or null. Equation (4) associates Boolean indicator variables ${z}_{\overline{r}}$ to active reaction fluxes, meaning reaction with non-null fluxes. Equation (5) defines specific Boolean indicator variables ${c}_{\overline{r}}$ for reaction fluxes corresponding to reactions in ${{{\mathcal{C}}}}ut$. These are the Boolean variables that are considered for subset-minimal solutions. The ${{{\mathcal{C}}}}ut$ reactions are the only reactions which flux is of interest: representing the actual reactions in MCSs. Equation (6) forbids the flux of two irreversible reactions issued from the split of a reversible one to be non-null. Equation (7) defines the domain of reaction fluxes ${v}_{\overline{r}}\forall \overline{r}\in {{{{\mathcal{R}}}}}_{dual}$ as real linear values, the domain of indicator variables ${z}_{\overline{r}}\forall \overline{r}\in {{{{\mathcal{R}}}}}_{dual}$ as Boolean logic values, and forces the target reaction flux to be non-null.

Taking all of these constraints and searching for subset minimal assignments of ${c}_{\overline{r}}$ to {*True*}, we obtain the MCSs disabling reaction targets ${{{\mathcal{T}}}}$. Information for dual metabolic network construction and the Minimal Cut Sets problem is summarized visually with a complete formalization for genome-scale metabolic models in Supplementary Fig. 7.

### Using *aspefm* to compute MCSs

The procedure is applied using *aspefm*, our Answer Set Programming (ASP) logic programming method^41^. The *aspefm* tool is distributed at https://github.com/maxm4/aspefm.

Given the compressed metabolic network in dual form, *aspefm* defines a logic program in Answer Set Programming able to enumerate all or only a subset of Minimal Cut Sets. ASP is a logic programming language, meaning it requires a declarative logic program to be defined as input. It is adapted for finding solutions to combinatorial problems thanks to its SAT-based solving. SAT refers to the well-studied Boolean satisfiability problem. The solver utilized in *aspefm* is state-of-the-art solver *clingo* extended with linear constraints through a modified *clingo*[LP] interface, with an IBM © *cplex* backend^72^.

Subset-minimal solutions are obtained with the set minimization heuristics of *clingo*, *aspefm*’s solver. No minimization of the size of the solutions searched is performed. An algorithmic amelioration was made to the *clingo*[LP] code. The algorithm for finding core conflicts from conflicting linear constraints was formerly implemented in recursive pure Python. We replaced it by *cplex*’s internal conflict refiner function. The code runs about 10-times faster on small and on larger models.

Another amelioration was made for direct enumeration of solutions with linear constraints, which are known to modify the solution space and thus its minimal solutions^73^. A solution checker was implemented, and it is called to verify the minimality and validity of solutions, in the case of MCSs with biomass as a target, it is a simple FBA call. This linear programming call should be very fast in computation time compared to the overall cost of the combinatorial exploration.

As with EFMs computation, additional constraints can be added to *aspefm*, only yielding a subset of all possible solutions. These constraints need to be expressed on the reactions from the compressed dual network, or undergo a conversion process if these relate to the original network.

### Answer set programming

Answer Set Programming (ASP) is a particular specification of logic programming. It is a widely used method for combinatorial problems: it has been applied to solve various biological problems, including problems related to reconstruction of constraint-based modelling networks^74,75^. The Answer Set Programming logic programming paradigm is oriented towards resolving constraint satisfaction problems, combinatorial optimization applications, and NP-hard problems in general. As with other logic programming methods, it defines declarative, automated reasoning programs in human-readable syntax, for which resolution is left to the computing machine. In ASP’s case, a set of solutions can be derived, and solutions are called *answer sets*. The language defines the so-called *stable models* semantics, where a model is a solution if and only if it is stable, and thus, answer sets are also called *stable models*. Answer sets are analogous to truth assignments of Boolean propositions, in regards to testing the satisfiability of a Boolean formula^76^. We recommend Lierler’s review as both an entry-level paper for experimented SAT modellers and a comprehensive look at the Answer Set Programming field^76^.

### Adding constraints to *aspefm*

Using *aspefm’s* input format, it is possible to add constraints to the computation of MCSs. Let us consider ${{{\mathcal{C}}}}ut$ the set of all reactions, and *c*_*r*_ Boolean variables representing if a reaction is cut or not. For example, we can express a size constraint, meaning that cut sets above a certain size *P* will not be computed:

8 $$\,{{\mbox{Card}}}\,\{{c}_{r}\,| \,{c}_{r}=1,\,r\in {{{\mathcal{C}}}}ut\} < P$$

Supposing we have a non-empty list of “unwanted reactions” of interest $U\subset {{{\mathcal{C}}}}ut$. The following negative Boolean constraint can be added:

9 $$\bigwedge \neg \,{c}_{r}\quad \forall r\in U$$

This will specify to the solver to only compute MCSs containing none of those reactions.

Supposing we have a non-empty list of “wanted reactions” of interest $W\subset {{{\mathcal{C}}}}ut$. The following positive Boolean constraint can be added:

10 $$\bigwedge {c}_{r}\quad \forall r\in W$$

This will specify to the solver to only compute MCSs containing all of those reactions. Note that since these positive Boolean inputs translate into adding linear constraints changing the solution space, our solution checker implemented in *aspefm* should be called.

### Retrieving information at the gene and protein level

Gene and protein level information was reviewed as part of the model curation process. Gene products defined in the SBML models were modified to all match UniProt or TrEMBL entries, in particular for *iYS854*. *iYS854* genes used new locus tags that yielded no UniProt query results, so genes were renamed to their old locus tags. UniProt entries were sought for using UniProt BLAST. For the gene products with no match in the str. JE2 strain, an homologue from a close *S. aureus* strain was used. In the process, new RDF annotations were added for future modellers. For both models, overall Boolean Gene-Protein-Reaction rules (GPRs) were simplified into canonical Disjunctive Normal Form (DNF), which helps with exhaustive enumeration of gene knock-outs from sets of reactions. Minimal sets of genes corresponding to the MCSs were computed using ASP logic programming with subset-minimization heuristics, as the problem can be expressed as minimal Boolean assignments to {*True*}.

Minimal sets of genes are non-trivial subset-minimal assignments to {*True*} respecting the GPRs Boolean formulae. However, as underlined in Machado et al.^39^, some gene products are ubiquitous, appearing in multiple reactions, meaning that the minimal sets of genes obtained from lethal MCSs of reactions might not necessarily be minimal in the number of genes to be knockout.

In order to better describe information at the gene or protein level, it is required that each reaction is associated to at least one gene. So, for transporter reactions that are not associated to genes, we added a dummy association, which in fact corresponds to the either backwards or forwards direction of that reaction, since reactions are split for MCSs computation. In practice, almost all reactions lacking genes are transporters, and forwards transporter reactions should also be assumed to be dependent on the presence of external metabolites in the medium. Some transporter reactions are also known to be spontaneous and annotated as such in their GPR association rules.

### Retrieving enzyme cuts targeting both bacteria for therapeutic action in human

To derive the sets of MCSs in common to both bacteria, reactions in MCSs were summed together, and MCSs from both models which had the same total mass balance equation were kept. The sets of MCSs in common were then converted into the corresponding minimal sets of genes with ASP logic programming, using GPRs derived from each bacterial model.

Protein structure models were retrieved using the AlphaFold entries^53^ associated to UniProt genes, and structure alignment was performed using algorithm FATCAT 2.0^77^. All retrieved genes using the model’s GPRs could be associated to UniProt entries, but very few of these entries had known crystallized structures in PDB. As such, only AlphaFold entries were compared together.

Enzyme targets with good interspecies protein structure alignments were tested for existence of human homologues in the UniProt database using their E.C. Number. For enzymes with no human homologues, enzymes were kept as possible targets. For enzymes which had human homologues, the corresponding AlphaFold structure predictions were retrieved, and further protein structure alignments were performed to check druggability.

All MCSs with common enzymatic function between the two bacteria were reported in Supplementary Dataset 2 as well as their FATCAT alignment scores. We further detail the procedure for protein structure alignments in the next two subsections.

### Interspecies protein structure alignments of enzyme targets

Protein structures with a FATCAT alignment RMSD above 3 Ångström were considered dissimilar between species (Supplementary Fig. 5). Excluded enzymes comprised Glutamyl-tRNA synthetase and reductase, and in particular, 3-dehydroquinate dehydratase, for which we confirmed through UniProt and InterPro that between *P. aeruginosa* and *S. aureus*, the enzymes had very different protein domains. Overall, we found that this threshold for scores of alignment between AlphaFold structure predictions was a helpful indicator of whether or not the proteins were similar between species.

Many MCSs would equivalently include exchange reactions or transporter reactions. Thus, we called these MCSs “growth medium dependent”. These are possible drug targets, but only in auxotrophic conditions, when amino acids are depleted from the medium. Although these cut sets might be challenging to use as therapeutic targets, we found them to be at least informative because if only the medium is depleted, then the enzymes become essential, and a drug targeting them would have an effect. These are also indicators of the main enzymes relating to a particular amino acid metabolism.

### Estimating quality of enzyme targets for therapeutic applications to humans

FATCAT alignments between the *S. aureus* and *P. aeruginosa* structures and all human homologues were performed. For each alignment, three categories were considered, based on FATCAT scores: “Structurally equivalent”: (*RMSD* < 3) and (*score* < 10^−6^), “Structurally similar”: (3 ≤ *RMSD* < 5) and (10^−6^≤ *score* < 10^−3^), “Structurally dissimilar”: (*RMSD*≥5) and (*score* ≥ 10^−3^). Then, only enzymes with strictly less than 50% human homologues which had “Structurally equivalent” alignments with both bacteria were kept as possible targets (Supplementary Fig. 5).

Most of the enzymes with human homologues were eliminated through this procedure, the analysis excluded thirteen potentially not targetable enzymes out of sixteen. Almost all excluded enzymes, for which alignments were classified as “Structurally equivalent”, had identical InterPro protein domains between human and bacteria, despite the large phylogenetic distance, thus making for dangerous therapeutic targets.

Finally, we decided to exclude the target nucleoside diphosphate kinase (ATP:UDP) (36.73% “Structurally equivalent”, 18 proteins similar out of 49 homologues), even though it scored high in terms of interspecies protein structure alignment, as we believe targeting this enzyme would not be viable in human cells. As a result, only two enzymes with human homologues were kept at the end of the analysis (Supplementary Fig. 2).

### Reporting summary

Further information on research design is available in the Nature Research Reporting Summary linked to this article.

### Supplementary information

Supplementary Material

Reporting summary

Dataset 1

Dataset 2

Dataset 3

## Data Availability

The datasets generated and analysed during the current study are provided in Supplementary Datasets and available from the corresponding author on request.

## Code availability

Code for the analysis is provided at https://github.com/maxm4/paSAmcs/.
