# Supplementary material for: Logic programming-based Minimal Cut Sets reveal consortium-level therapeutic targets for chronic wound infections
Source: NPJ Syst Biol Appl. 2024 Apr 2;10:34. doi: 10.1038/s41540-024-00360-6 (PMC10987626; doi:10.1038/s41540-024-00360-6)
Supplement: Supplementary file 1 — Supplementary Material [file 41540_2024_360_MOESM1_ESM.pdf]

Logic programming-based Minimal Cut Sets reveal consortium-level therapeutic targets for chronic wound infections

Maxime Mahout, Ross Carlson, Laurent Simon, Sabine Peres

Computation times of each tool for single-species MCSs of size three or fewer

We run the three MCSs computation tools *aspefm*, *CNApy* and *CoBAMP* on the single-species models of *P. aeruginosa* and *S. aureus* of size three or fewer. In contrast to the results for the consortium-level analysis with a size limit of 16 and a time limit of 1.5, on single-species *CoBAMP* performed the best with its iterative method, getting all MCSs without any issues and under 2 hours and 30 min.

In contrast, both *CNApy* and *aspefm* presented cases where they overestimated the number of MCSs. In *aspefm*'s case, this was apparent on the *S. aureus* model, with occasional solver numerical issues. In *CNApy*'s case, eventually some MCSs of *S. aureus* were skipped and the number of MCSs was also underestimated. It is possible these numerical issues come from biomass composition or network compression. For *aspefm*, invoking our extension checking the validity of solutions as previously mentioned in Methods completely solves the problem and allows enumeration to be successfully performed. Additionally, *aspefm* struggles to conclude after all solutions were found, so a fixed limit on the number of conflicts should be added to programatically terminate computation.

In conclusion, while non-ordered MCSs enumeration methods such as *CNApy* and *aspefm* show very promising results for larger-sized MCSs, they do so with possible losses of performance in regards to enumeration of smaller-sized MCSs, which iterative methods such as *CoBAMP* handle consistently well. Further benchmarks should be performed on particular networks to assess the cases where each method performs best.

P. aeruginosa	*aspefm*	*CNApy*	*CoBAMP*
Computation time	1h 18 min	1h 48 min	2h 02 min
Number of solutions	583/583	631/583	583/583
S. aureus	*aspefm*	*CNApy*	*CoBAMP*
Computation time	4h 56 min	5h 44 min	2h 28 min
Number of solutions	944/938	934/938	938/938

Supplementary Table 1: Benchmark of computation times of each tool for the computation of single-species MCSs of size three or fewer. *aspefm* times do not include network compression and decompression, nor the time required to conclude after all correct solutions were found.

Possible metabolite uptakes from the medium

P. aeruginosa medium metabolite exchanges	*S. aureus* medium metabolite exchanges	Consortium medium metabolite exchanges
EX_ala_L_e EX_arg_L_e EX_asn_L_e EX_asp_L_e EX_ca2_e EX_cbl2_e EX_cit_e EX_cl_e EX_co2_e EX_cobalt2_e EX_csn_e EX_cu2_e EX_cys_L_e EX_fe2_e EX_fe3_e EX_glc_D_e EX_gln_L_e EX_glu_L_e EX_gly_e EX_h2o_e EX_h_e EX_his_L_e EX_ile_L_e EX_inost_e EX_k_e EX_lac_D_e EX_leu_L_e EX_lys_L_e EX_met_L_e EX_mg2_e EX_mn2_e EX_mobd_e EX_na1_e EX_nh4_e EX_o2_e EX_phe_L_e EX_pi_e EX_pro_L_e EX_ser_L_e EX_so4_e EX_thm_e EX_thr_L_e EX_trp_L_e EX_tyr_L_e EX_ura_e EX_val_L_e EX_zn2_e	EX_4abz_e EX_ade_e EX_ala_L_e EX_arg_L_e EX_asn_L_e EX_asp_L_e EX_btn_e EX_ca2_e EX_cbl2_e EX_cl_e EX_co2_e EX_cobalt2_e EX_csn_e EX_cu2_e EX_cys_L_e EX_fe2_e EX_fe3_e EX_fol_e EX_glc_D_e EX_gln_L_e EX_glu_L_e EX_gly_e EX_gua_e EX_h2o_e EX_h_e EX_his_L_e EX_ile_L_e EX_inost_e EX_k_e EX_lac_D_e EX_leu_L_e EX_lys_L_e EX_met_L_e EX_mg2_e EX_mn2_e EX_mobd_e EX_na1_e EX_nac_e EX_nh4_e EX_o2_e EX_phe_L_e EX_pi_e EX_pnto_R_e EX_pro_L_e EX_pydam_e EX_pydx_e EX_ribflv_e EX_ser_L_e EX_so4_e EX_thm_e EX_thr_L_e EX_trp_L_e EX_tyr_L_e EX_ura_e EX_val_L_e EX_zn2_e	EX_4abz_e EX_ade_e EX_ala_L_e EX_arg_L_e EX_asn_L_e EX_asp_L_e EX_btn_e EX_ca2_e EX_cbl2_e EX_cit_e EX_cl_e EX_co2_e EX_cobalt2_e EX_csn_e EX_cu2_e EX_cys_L_e EX_fe2_e EX_fe3_e EX_fol_e EX_glc_D_e EX_gln_L_e EX_glu_L_e EX_gly_e EX_gua_e EX_h2o_e EX_h_e EX_his_L_e EX_ile_L_e EX_inost_e EX_k_e EX_lac_D_e EX_leu_L_e EX_lys_L_e EX_met_L_e EX_mg2_e EX_mn2_e EX_mobd_e EX_na1_e EX_nac_e EX_nh4_e EX_o2_e EX_phe_L_e EX_pi_e EX_pnto_R_e EX_pro_L_e EX_pydam_e EX_pydx_e EX_ribflv_e EX_ser_L_e EX_so4_e EX_thm_e EX_thr_L_e EX_trp_L_e EX_tyr_L_e EX_ura_e EX_val_L_e EX_zn2_e

Supplementary Table 2: Listing of metabolite exchanges where uptake is possible as defined by the medium for: *a) b)* each single-species bacterial model, *c)* the consortium model.

Possible interspecies metabolite exchanges in the consortium model

Metabolites secreted by *P. aeruginosa* when a part of the consortium model	Metabolites secreted by *S. aureus* when a part of the consortium model
M_2dhglcn_e M_4abut_e M_ac_e M_acac_e M_acgam_e M_acon_C_e M_adn_e M_akg_e M_ala_L_e M_alltn_e M_arg_L_e M_asn_L_e M_asp_L_e M_bhb_e M_cit_e M_co2_e M_cytd_e M_dad_2_e M_dad_5_e M_dcyt_e M_fru_e M_fum_e M_glcn_e M_glu_L_e M_gly3p_e M_gly_e M_glyc_e M_glyclt_e M_h2o_e M_h_e M_his_L_e M_hxan_e M_ile_L_e M_ins_e M_itacon_e M_lac_D_e M_leu_L_e M_lys_L_e M_mal_L_e M_nh4_e M_no2_e M_o2_e M_orn_e M_phe_L_e M_pi_e M_ppa_e M_ppi_e M_pqs_e M_pro_L_e M_ptrc_e M_pyo_e M_pyr_e M_rib_D_e M_ser_L_e M_succ_e M_thr_L_e M_thymd_e M_trp_L_e M_tyr_L_e M_ura_e M_urcan_e M_urea_e M_uri_e M_val_L_e M_xan_e	M_2obut_e M_3gmp_e M_ac_e M_acald_e M_acgam1p_e M_acgam_e M_acglu_e M_actn_R_e M_ade_e M_aglaa_e M_arg_L_e M_asp_L_e M_btd_RR_e M_butACP_e M_cellb_e M_citr_L_e M_co2_e M_dad_2_e M_dcaACP_e M_dcyt_e M_dgsn_e M_dhap_e M_din_e M_etoh_e M_f6p_e M_fe3_e M_for_e M_forglu_e M_fru_e M_g6p_B_e M_gam6p_e M_gam_e M_glu_L_e M_glyald_e M_glyc_e M_glyclt_e M_h2o_e M_h_e M_his_L_e M_hom_L_e M_hxan_e M_ile_L_e M_ins_e M_istfrnA_e M_istfrnB_e M_lac_D_e M_lac_L_e M_leu_L_e M_lys_L_e M_met_L_e M_mnl1p_e M_mnl_e M_nh4_e M_no2_e M_no3_e M_orn_e M_phe_L_e M_pi_e M_ppi_e M_ptrc_e M_pyr_e M_rib_D_e M_ribflv_e M_ser_L_e M_stfrnA_e M_stfrnB_e M_thym_e M_trp_L_e M_tyr_L_e M_ura_e M_urea_e M_val_L_e M_xan_e
**Metabolites secreted by *P. aeruginosa* possibly taken up by *S. aureus***	**Metabolites secreted by *S. aureus* possibly taken up by *P. aeruginosa***
M_ac_e M_acgam_e M_akg_e M_ala_L_e M_arg_L_e M_asn_L_e M_asp_L_e M_co2_e M_cytd_e M_dad_2_e M_dcyt_e M_fru_e M_fum_e M_glcn_e M_glu_L_e M_gly_e M_glyc_e M_h2o_e M_h_e M_his_L_e M_hxan_e M_ile_L_e M_ins_e M_lac_D_e M_leu_L_e M_lys_L_e M_mal_L_e M_nh4_e M_no2_e M_o2_e M_orn_e M_phe_L_e M_pi_e M_pro_L_e M_ptrc_e M_pyr_e M_rib_D_e M_ser_L_e M_succ_e M_thr_L_e M_thymd_e M_trp_L_e M_tyr_L_e M_ura_e M_urea_e M_val_L_e M_xan_e	M_ac_e M_acgam_e M_arg_L_e M_asp_L_e M_co2_e M_fru_e M_glu_L_e M_glyc_e M_h2o_e M_h_e M_his_L_e M_hxan_e M_ile_L_e M_ins_e M_lac_D_e M_lac_L_e M_leu_L_e M_lys_L_e M_met_L_e M_mnl_e M_nh4_e M_no2_e M_no3_e M_orn_e M_phe_L_e M_pi_e M_ptrc_e M_pyr_e M_rib_D_e M_ser_L_e M_trp_L_e M_tyr_L_e M_ura_e M_urea_e M_val_L_e M_xan_e

Supplementary Table 3: Listing of metabolites secreted by *P. aeruginosa* and *S. aureus* in the consortium model, including metabolites available for cross-feeding: metabolites secreted by a bacterium and possibly taken up by the other.

Supplementary figures

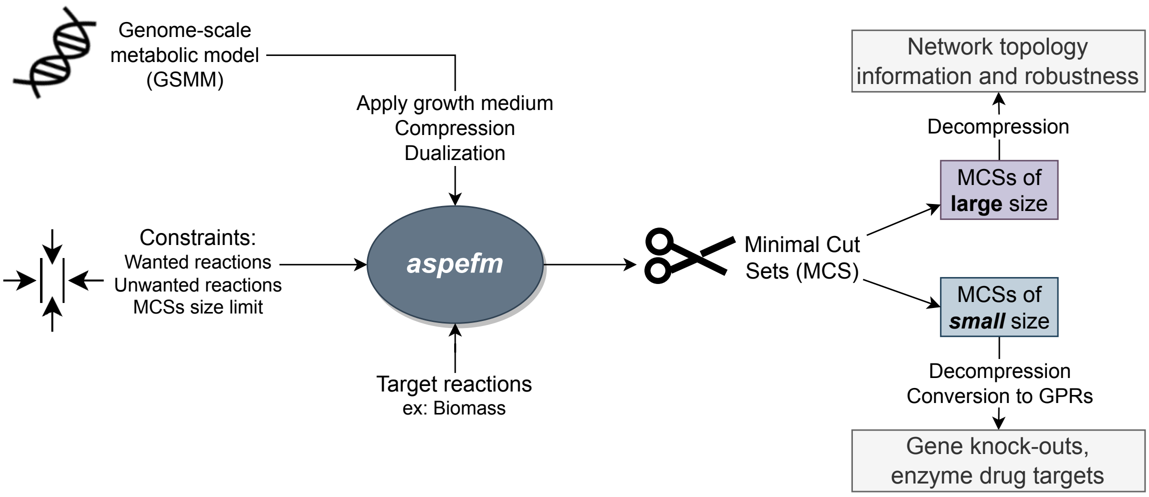

Supplementary Figure 1: *aspefm* framework applied on the GSMMs of *Staphylococcus aureus*, *Pseudomonas aeruginosa*, and on the consortium model.

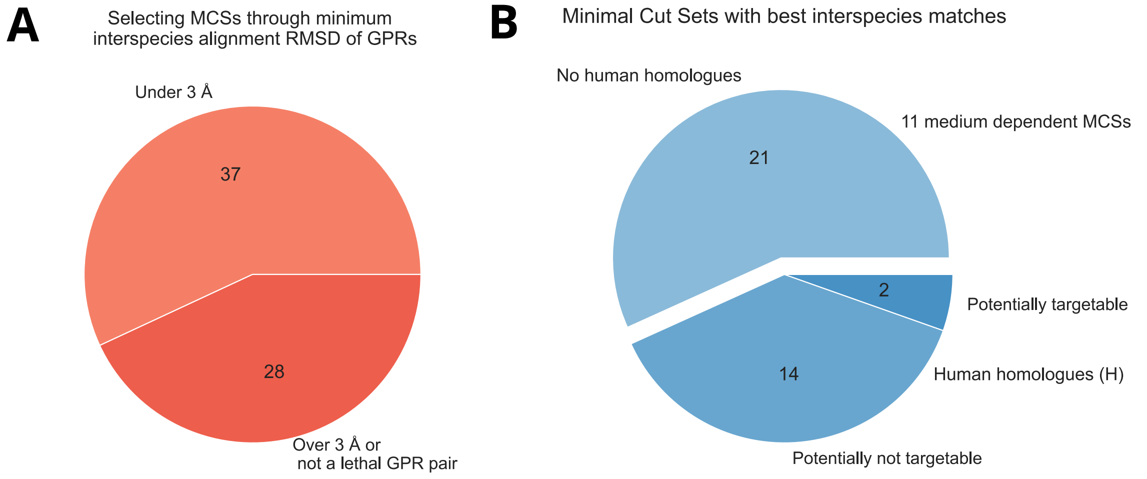

Supplementary Figure 2: **A**: Analysis of GPRs and RMSD scores of interspecies protein structure alignments for the enzyme targets from GPRs, corresponding to the 65 MCSs. **B**: Summary of the search of human homologues, leaving 23 'good' potential targets, including 11 medium dependent ones, out of the 37 considered targets.

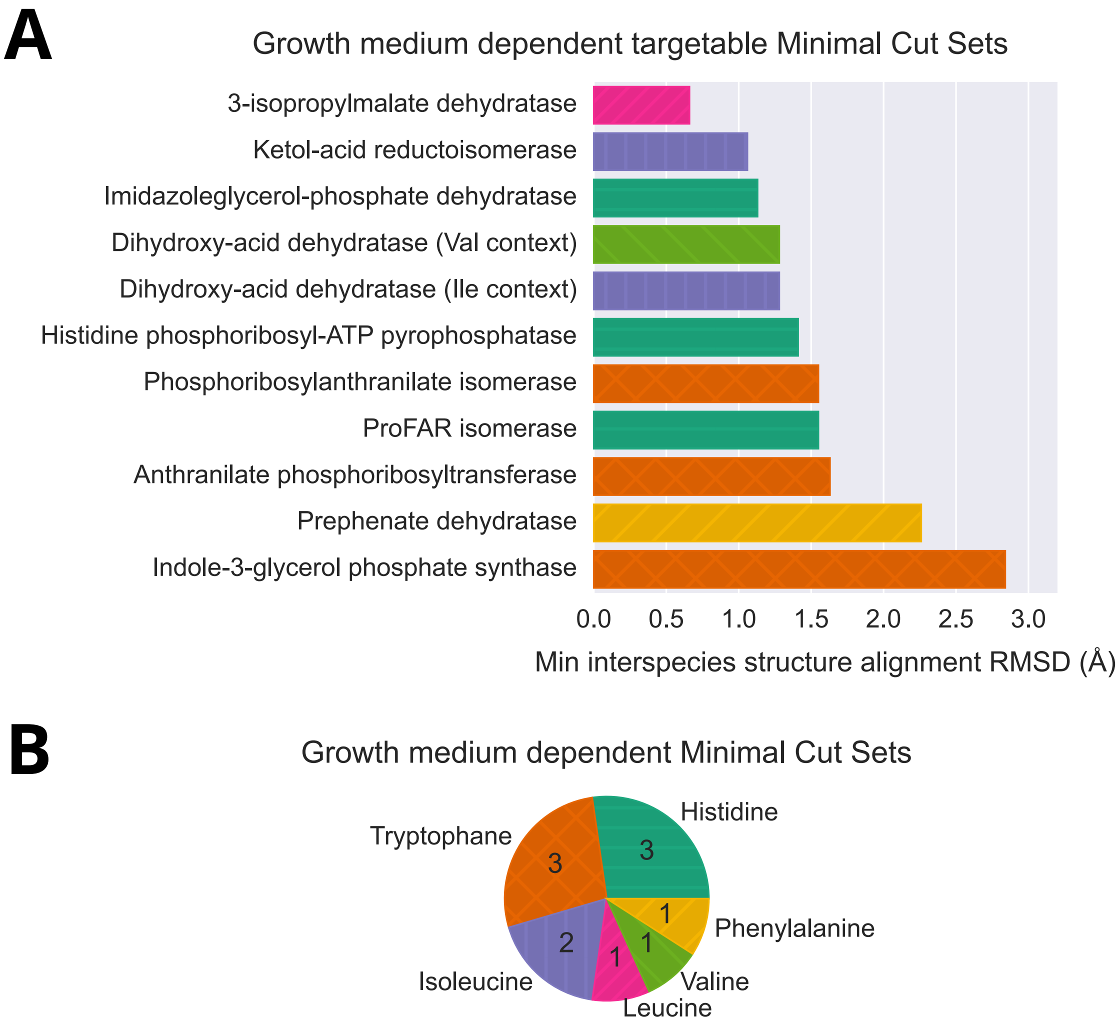

Supplementary Figure 3: A: Enzyme targets dependent on the presence of amino acids in the growth medium. Bars heights are minimum Root Mean Square Deviation (RMSD) of atom positions, measured in Ångström, resulting from protein structural alignment of GPR pairs. **B**: Associated amino acids and pie distribution for each MCSs in **A**.

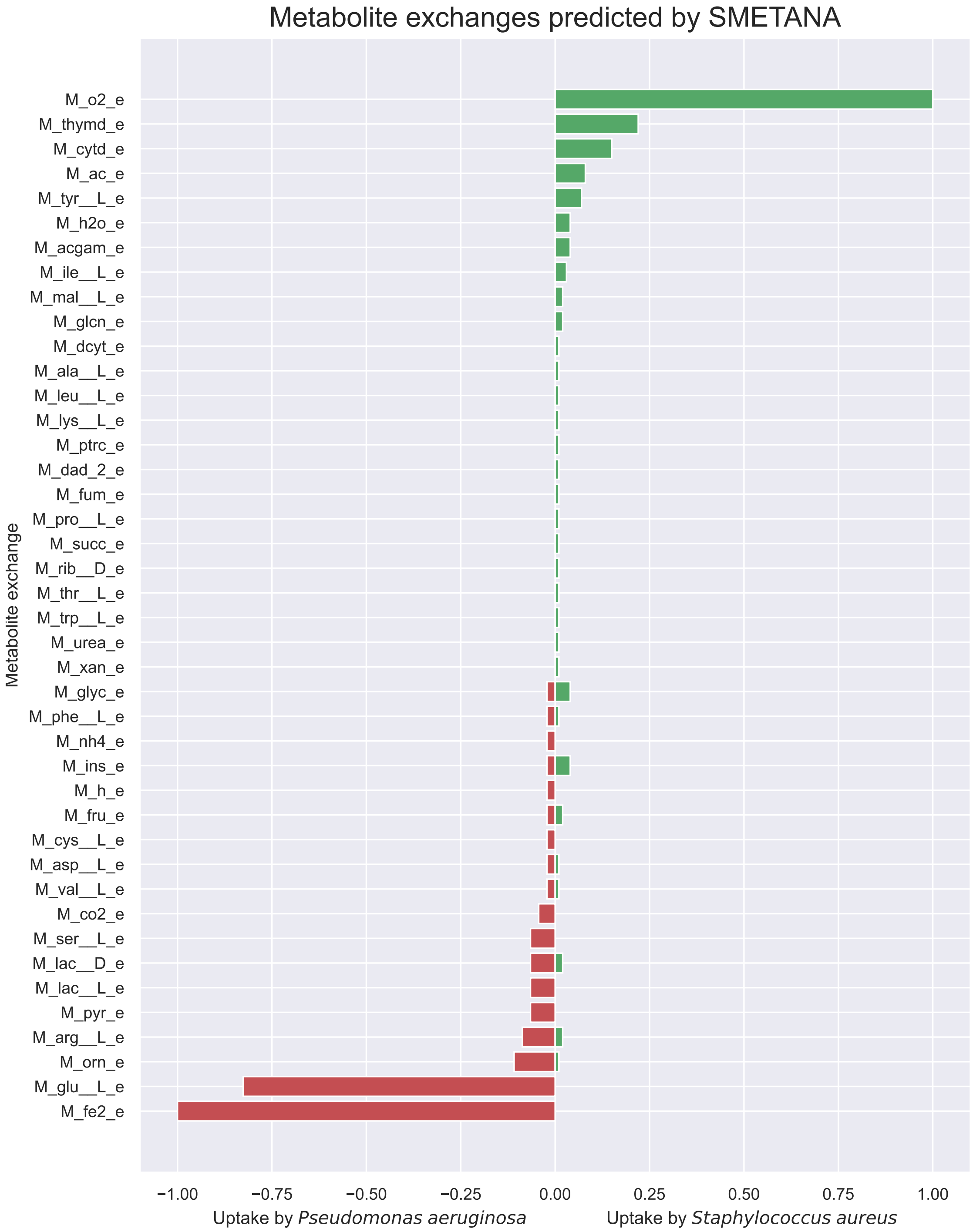

Supplementary Figure 4: Estimated potentials for metabolite exchanges between the two bacteria according to SMETANA. Abscissae are SMETANA scores, multiplied by -1 when S.A. is the donor instead of P.A.

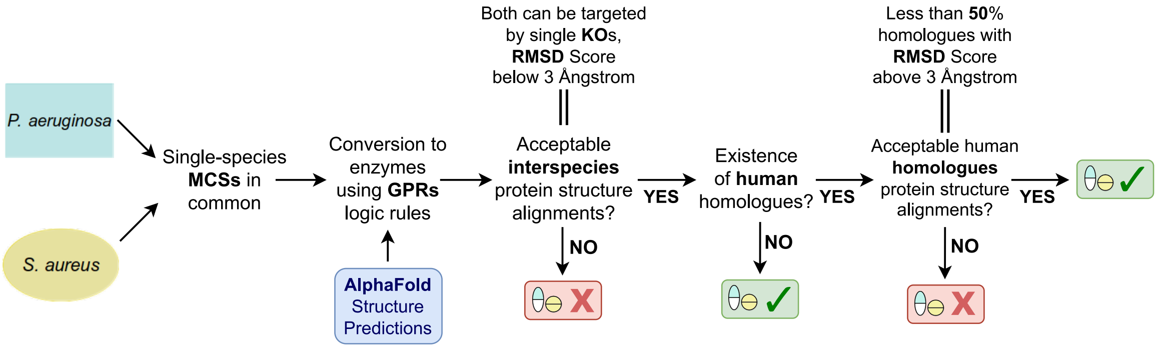

Supplementary Figure 5: Search procedure for finding new therapeutic targets using protein structure alignments of AlphaFold predictions.

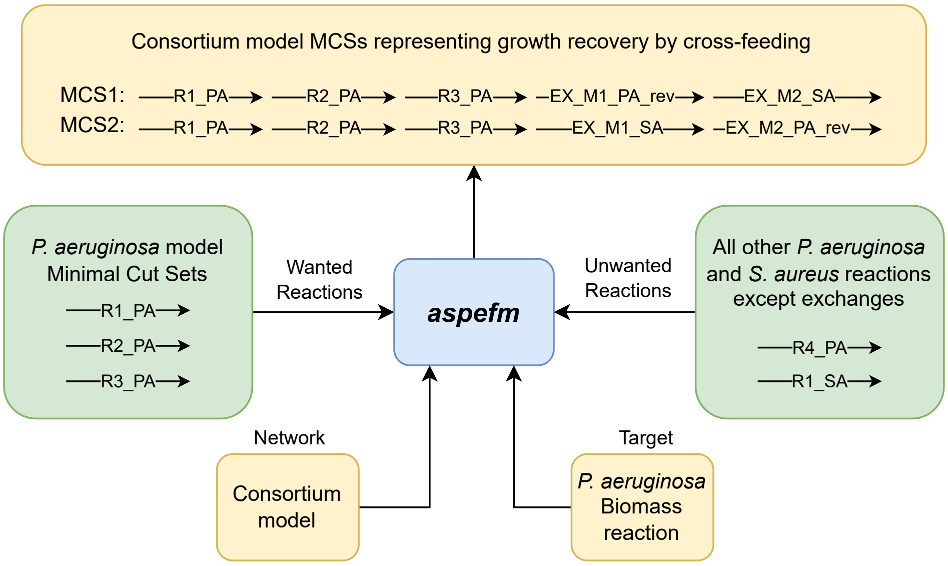

Supplementary Figure 6: Procedure for computing MCSs revealing metabolite exchanges with *aspefm*, from the sets of lethal MCSs of either bacteria with growth recovery on the consortium model (illustrated for *P. aeruginosa*). Exchange reactions are denoted by "**EX**". Backwards direction of a reaction is denoted by "rev". MCSs with growth recovery on the consortium model are selected as "wanted reactions" constraints, while all other metabolic reactions except exchanges usable for cross-feeding are set as "unwanted reactions". After running *aspefm*, output consortium model MCSs represent growth recovery by cross-feeding of external metabolites. Displayed on the figure, cross-feeding of external metabolites M1 and M2 complement lethality of cutset {R1, R2, R3} of *P. aeruginosa*, thus the new cut sets obtained on the consortium model with *P. aeruginosa*'s biomass reaction as target have: {R1, R2, R3} as base, then either consumption of M1 by PA or production of M1 by SA needs to be cut, and then either consumption of M2 by PA or production of M2 by SA needs to be cut (two examples of such cut sets shown out of four).

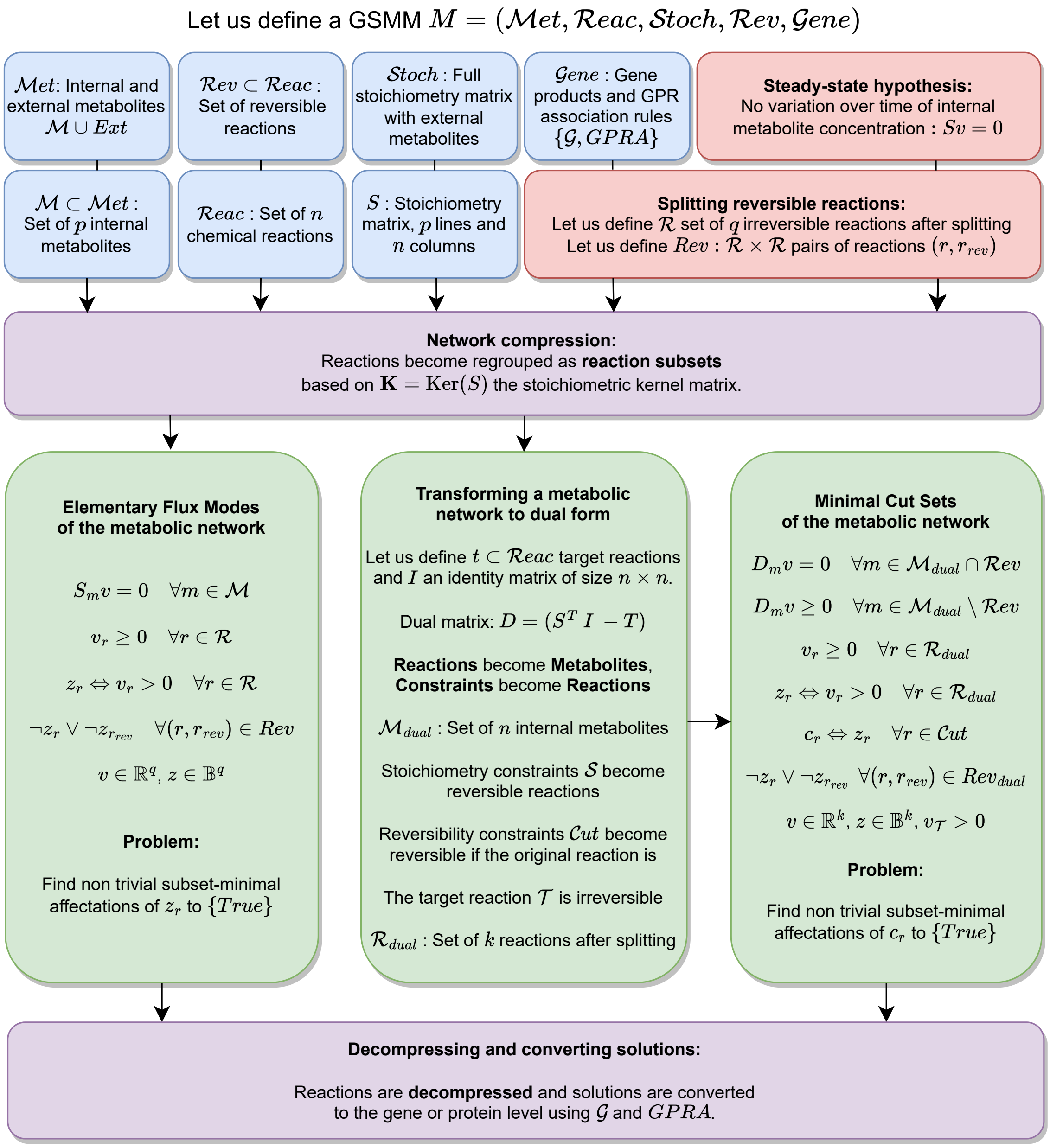

Supplementary Figure 7: Diagram of the problem of computing MCSs and its formulation in a comprehensive view. Minimal Cut Sets are expressed as a dual problem to Elementary Flux Modes. To make use of GSMMs, network compression is required, and solutions are converted back to genes using GPRs.
